# Liquid Biopsy and Primary Brain Tumors

**DOI:** 10.3390/cancers13215429

**Published:** 2021-10-29

**Authors:** Robert H. Eibl, Markus Schneemann

**Affiliations:** 1 c/o M. Schneemann, Department of Internal Medicine, Hospitals of Schaffhausen, 8208 Schaffhausen, Switzerland; 2 Department of Internal Medicine, Hospitals of Schaffhausen, 8208 Schaffhausen, Switzerland

**Keywords:** liquid biopsy, brain tumor, glioblastoma, glioma, medulloblastoma, circulating tumor cell—CTC, circulating tumor (ct)DNA, extracellular vesicle, microRNA—miR, biomarker

## Abstract

**Simple Summary:**

Despite a substantial increase in publications in recent years, liquid biopsy from blood, cerebrospinal fluid (CSF), and other body fluids is usually not routinely used in cancer diagnostics and tumor monitoring. In this regard, brain tumors represent an additionally challenging group of tumors due to the blood–brain barrier as a potential suppressor of migrating tumor cells and their property of rarely metastasizing via the blood. Surprisingly, however, circulating tumor cells (CTCs) have been found in 20% of glioblastoma patients, which may allow for monitoring of tumor progression and response to therapies based on the genetic profiling of such tumors. Genetic biomarkers from CTC, circulating tumor DNA (ctDNA), extracellular vesicles, and microRNA (miRNA) are discussed. Here, we review the recent developments and future potential of liquid biopsy in brain tumors.

**Abstract:**

Two decades of “promising results” in liquid biopsy have led to both continuing disappointment and hope that the new era of minimally invasive, personalized analysis can be applied for better diagnosis, prognosis, monitoring, and therapy of cancer. Here, we briefly highlight the promises, developments, and challenges related to liquid biopsy of brain tumors, including circulating tumor cells, cell-free nucleic acids, extracellular vesicles, and miRNA; we further discuss the urgent need to establish suitable biomarkers and the right standards to improve modern clinical management of brain tumor patients with the use of liquid biopsy.

## 1. Introduction

Two of the most prominent neurosurgeons of the 20th century, Harvey Cushing for the first half and Gazi Yasargil for the second half, were named the “men of the century” in their field; Cushing significantly increased the survival rate of brain tumor operations, whereas Yasargil applied microsurgery to remove tumors in the brain at a new level of precision using binocular microscopy [1,2]. Both contributed to the histological classification of these infrequent tumors (2% of all cancers). More recent methods in cell and molecular biology included the experimental search for the cell of origin with new tumor models and oncogene transfer [3,4,5], which is also addressed in all cancers by the concept of cancer stem cells [6]. Since 1979, several updated editions of the WHO classification of tumors of the nervous system appeared; histological typing by light microscopy on hematoxylin and eosin-stained sections allowed for characterization and grouping these tumor entities, typically supported by immunohistochemical detection of lineage-associated protein markers. Later on, spectacular advances in genetics allowed further analysis of tumor development, including loss of heterozygosity (LOH) and specific detection of mutations in oncogenes and tumor suppressor genes. Genetic analysis of tumor samples revealed correlations of tumor entities with specific mutations, such as p53 [7,8,9,10], which complemented the pure histology of brain tumor biopsies. The newest WHO classification of brain tumors from 2016 allows a thorough characterization of new subsets of brain tumors, not only by their histology but also, independently, by their molecular features, which revolutionized brain tumor diagnostics [11]. Today, data on molecular genetics can outperform or complement pure histology; several tumor entities of glioblastoma, medulloblastoma, diffuse midline glioma, and ependymoma can now further be characterized by their genetic status as isocitrate dehydrogenase (IDH)-wildtype, IDH-mutant, histone H3 K27M-mutant, rearrangement during ependymal cell differentiation (RELA) fusion-positive, WNT-activated, and sonic hedgehog (SHH)-activated. Twenty-five years after one of the authors found the first p53 mutation in a primary medulloblastoma [7], when others were able to detect similar mutations in cell lines, but not in primary medulloblastomas [12], molecular diagnostics became a state-of-the-art method in tissue biopsies of brain tumors. For example, childhood medulloblastomas can currently be seen as four different subsets, allowing different prognosis and treatment [11]. Gliomas represent a major proportion of brain tumors. Half of these are glioblastoma multiforme (GBM, WHO grade IV), the most severe tumor type with only about 1 year of survival time. Glial fibrillary acidic protein (GFAP) is widely expressed in astroglial and neural stem cells in the brain, and it also serves as a major marker in immunohistology of astroglial tumors, such as glioblastomas. In a pilot study, this protein was also detectable in the serum of patients with astrocytic tumors, but not in control patients [13]. O^6^-Methylguanine-DNA methyltransferase (MGMT) is relevant for DNA repair induced by alkylating agents. MGMT promoter methylation typically causes reduced levels of MGMT in glioblastoma cells and makes them susceptible to alkylating agents [14,15,16]. IDH1/2 are enzymes that protect against oxidative damage in the cell. IDH mutants impair DNA repair, increasing chemosensitivity, and are correlated with longer overall survival (OS) and progression-free survival (PFS). Several alterations of the epidermal growth factor receptor (EGFR) are known for glial tumors: mutations, overexpression, and variant expression, which often lead to a ligand-independent, oncogenic function. Variable DNA deletions can result in loss of exons 2–7 of the EGFR gene, resulting in the splice variant EGFRvIII, conferring resistance to chemotherapy [17]. MGMT, IDH, and EGFR are clinically relevant examples of diagnostic and prognostic markers used in tissue biopsies; it is reasonable to apply this knowledge by evaluating these and other potential markers in less invasive liquid biopsies. In the near future, perhaps within 5 years, liquid biopsy may also add its emerging findings of genetic profiling for accurate diagnosis, monitoring tumor status, and treatment response with respect to new therapies, thus improving prognosis and clinical management of brain tumor patients. Liquid biopsy offers a better analysis with the advantage of posing a lower risk in obtaining material from the tumor. An increasing number of original papers, as well as many reviews, have outlined the challenges in finding and applying different methods of liquid biopsy for the most common tumors. So far, breast cancer and prostate and colorectal carcinomas are the frontrunners in clinical applications. The limited number of reviews on liquid biopsy and brain tumors, often restricted to only specific subtypes [18,19,20,21,22,23,24,25], demonstrates that brain tumors are still out of the main focus of liquid biopsy applications; here, we summarize the fundamental challenges and recent developments, as well as provide our view of how to resolve these.

## 2. Liquid Biopsy

**Figure 1:**
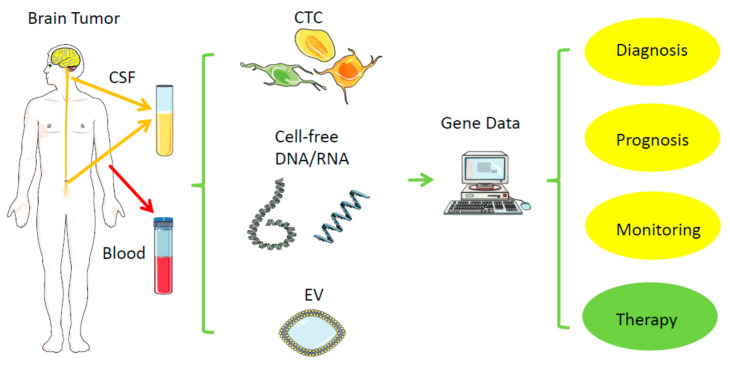
Liquid biopsy. Distant from the original brain tumor, samples from blood and cerebrospinal fluid (CSF) can typically serve as a low-risk source of tumor-derived nucleic acids (RNA, DNA) for further analysis. CSF—cerebrospinal fluid; EV—extracellular vesicle; CTC—circulating tumor cell. Created/modified with https://smart.servier.com (accessed on 8 August 2021), licensed under Creative Commons Attribution 3.0 Unported License (https://creativecommons.org/licenses/by/3.0/ (accessed on 8 August 2021)).

Direct biopsies obtain tissue material from the primary tumor, either via neurosurgical removal of all or most parts of a tumor or via stereotactic tissue biopsy. In contrast, a liquid biopsy uses body fluids collected distant to the brain tumor, such as venous blood from the arm or cerebrospinal fluid (CSF) via lumbar or cisternal puncture (Figure 1, Table 1) [26]. In general, probes from urine, saliva, ascites, bronchial fluid, or vitreous liquid can also serve as material for liquid biopsy, but mainly for other tumors. Within these biofluids, the relevant tumor-derived nucleic acids can be found in different compartments: (1) in single or clustered, intact circulating tumor cells (CTCs), (2) in subcellular parts derived from the tumor, such as extracellular vehicles (EVs), or (3) in even cell-free nucleic acids (cfDNA, RNA). Due to the location, tissue biopsies from brain tumors represent a usually much higher risk for complications than from most other tumors; hemorrhage or brain swelling can harm healthy parts of the brain and endanger life. Repeated tissue biopsies of the brain for follow-ups are difficult; thus, it is tempting to replace or complement them with a less risky procedure, as long as there is high specificity and acceptable sensitivity. Replacing a surgical tissue biopsy from a regrowing brain tumor by obtaining blood or CSF for liquid biopsy instead, while still getting the relevant information of the response to a therapy even more quickly and cheaply, would significantly reduce the individual risk for the patient, especially those with severe comorbidities. Tissue biopsies may miss a relevant part of a tumor, which liquid biopsy may detect, and, vice versa, liquid biopsy may also not represent the whole primary tumor, but represent a more migratory and aggressive part, thus potentially having some advantage or additional information over classical tissue biopsy.

### 2.1. Circulating Tumor Cells—CTCs

Circulating tumor cells (CTCs) are cells derived from a tumor which enter the bloodstream and other body fluids, e.g., the CSF. Unfortunately, they are extremely rare for almost all cancers; so far, a widely accepted standard method for identifying and collecting CTCs is still lacking. Since brain tumors do not usually metastasize via the bloodstream, it was quite a surprise to find CTCs in 20–40% of glioblastoma patients [27,28,29,30,31,32]. In carcinoma, clusters of tumor cells are increasingly associated with easier metastatic spread compared to single CTCs; similar clusters can be found in glioblastomas [31], supporting the idea that glioblastoma-derived CTCs can cross the blood–brain barrier as clusters of cells, although they almost never establish metastases. Applying the seed-and-soil hypothesis from Paget from 1889, they may have the ability to spread (seed), but cannot find the right target tissue or endothelium (soil), where they can egress, survive, and grow [33]. The limited overall survival time of the patients may also prevent micrometastases from growing larger after seeding. Different approaches exist to isolate CTCs for many tumor entities. To isolate CTCs from carcinoma, an elegant method uses epithelial cell adhesion molecule (EpCAM) as a surface marker, which is exclusively expressed on epithelia and epithelial-derived neoplasms; since EpCAM is not expressed on leukocytes, it can be used to selectively accumulate CTCs. In contrast, EpCAM is not expressed on brain tumor cells and, therefore, cannot be used as a selection marker for these cancers. Currently, no adequate surface marker for brain tumor CTCs has been established. Unfortunately, other isolation methods, which use the bigger size of tumor cells, are still less efficient. However, in at least 50% of the cases, CTCs from brain tumors can in fact be isolated from the blood or CSF. These can be extremely informative for molecular diagnostics with subclassification of tumors, as well as prognosis and repeated monitoring of progression and therapy resistance. The specificity of mutations found in CTCs is very high, but low sensitivity remains a big challenge, i.e., not all liquid biopsies from brain tumor samples allow the isolation of CTCs. It is noteworthy that brain tumor CTCs are easier to collect from CSF than from blood. Interestingly, cisternal puncture appears to give even better results than lumbar puncture, although the CSF is moving downward and is fully exchanged 3–5 times per day. Interestingly, circulating epithelial, i.e., noncancerous, cells have also been found in benign, inflammatory bowel diseases [34]; this points to the need for molecular characterization of cells obtained for CTC testing. Collection of CSF is usually performed easily, with a rare risk of damage to the brain. Major challenges for CTCs in brain tumors are the low sensitivity and the need for standardization, e.g., the amount of material and the source (blood, serum, plasma, or CSF from lumbar or cisternal puncture). If possible, cisternal puncture appears to be superior, but this procedure is not as easily performed as a lumbar puncture. For blood samples, 7.5 mL may become a standard for some, although higher amounts of blood increase the chance of finding CTCs, which is why others recommend over 30 mL. Due to these limitations, CTCs are only useful for a few patients with brain tumors. The collection of a sufficient amount of CSF may appear to be an additional challenge for children with brain tumors, such as medulloblastomas, but usually not for adults; it is also possible to use a previously implanted shunt from the ventricle to collect CSF. In carcinomas, the number of CTCs was correlated with time of survival, i.e., patients with more than a certain number died early, while others survived up to 10 times longer [35].

### 2.2. Cell-Free DNA and Circulating Tumor DNA

In 1948, two French scientists found nucleic acids in human blood [36], but only 60 years later was this finding applied to detect specific mutations found in colon cancer [37]. In cancer patients, circulating tumor DNA (ctDNA) represents only a fraction of the total cell-free DNA (cfDNA). In a xenograft model, the principal fragment length of human glioblastoma ctDNA was typically shorter than the background rat cfDNA, 134–144 bp vs. 167 bp, respectively [38]. Such distinct differences between normal cfDNA and tumor-derived ctDNA allow selection for the shorter cfDNA to increase sensitivity.

Earlier studies showed that specific mutations well known from tissue biopsies can also be detected in serum [39,40,41,42], in plasma [43,44,45], or in both [40]. More recent studies changed from searching for single, specific mutations to sequencing panels of tumor-related genes in plasma [46,47,48]. CSF showed a significantly higher sensitivity than serum or plasma in such multigene assays [49,50,51,52,53]. With next-generation sequencing (NGS), it was shown that CSF-derived ctDNA represented genomic alterations of brain tumors better than blood-derived ctDNA [54]. The first detection of frequent and important histone H3 mutations in CSF in children with usually unresectable midline glioma supports the clinical utility of such an approach [55], since CSF is more safely accessible than tissue biopsy from the brainstem or thalamus.

Despite a high specificity to detect tumor-associated mutations in ctDNA from blood or CSF, variable sensitivity limits the use of ctDNA for routine clinical applications [53,55]. Independent of tumor size, entity, and grading, a close location to a neighboring CSF reservoir correlated with a higher sensitivity to detect the ctDNA of medulloblastomas, ependymomas, and high-grade gliomas [53], although, surprisingly, not all tumors (ependymoma, low-grade glioma) abutting CSF space were detectable in this way. However, under certain conditions, liquid biopsy can be beneficial for some patients in order to (1) differentiate between pseudoprogression and real tumor progression, (2) monitor tumor response after surgery, chemotherapy, or radiation therapy, or (3) monitor tumor relapse before image diagnostics.

Over two decades, several mutations and detection methods from blood and CSF have evolved (more or less) chronologically (Table 2). The initial focus was on methylation-specific PCR of the MGMT promoter, p16, DAPK, RASSF1A, p73, PTEN promoter, p15INK4B, and p14ARF, as well as the LOH of 10q, 1p, and 19q, and sequencing specific mutations of PTEN, IDH1/2, EGFR, TP53, PIK3CA, EPHB1, telomerase reverse transcriptase (TERT), ANK, FTH1, OR51D1, NF2, AKT1, Met, ATRX, H3F3H, HIST1H3B, BRAF, JAK2, NF1, NA-RAS, GNAS, ATM, 1P19Q, and CIC [39,40,41,42,43,44,45,52,53,56,57,58]. More recent studies analyzed a number of genes by sequencing panels of genes from 54–70 genes up to whole-genome analysis [44,47,51]. Some of these typical mutations known from surgical biopsies are also relevant for therapeutic decisions [15,16]. Detection limits vary, and sensitivity appears to be better from CSF. Currently, a standard to use ctDNA in brain tumors needs to be established.

### 2.3. MicroRNA—miRNA—miR

MicroRNAs (miRNA, miR) are only 20–24 nucleotides long, i.e., very small, noncoding RNA molecules derived from just 1% of the whole genome. They are strongly involved in regulation of the stability and translation of mRNA in health and disease. Although first found in 1993 in the nematode *Caenorhabditis elegans* [61], the potential biological effects of up to 1900 miRNAs in humans are not completely understood. Many seem to play a role in tumor biology, angiogenesis and immunology and some can be considered as promising prognostic factors or as potential therapeutic targets in glioblastoma (Table 3) [56].

Most of the over 20 studies on miRNAs in gliomas showed variable, reasonable degrees of sensitivity and specificity, both often over 80% to 90%. The miRNAs relevant for brain tumors are often upregulated with a worse prognosis, but can also be downregulated compared to others: miR-10b, miR-15b, miR-15b-5p, miR-16-5p, miR-19b-3p, miR-20a-5b, miR-20a-5p, miR-21, miR-23a, miR-29, miR-106a-5p, miR-125, miR-128, miR-125, miR-125b, miR-128, miR-130-3p, miR-133a, miR-145-5p, miR-150, miR-181b-5p, miR-182, miR-182-5p, miR-197, miR-205, miR-208a-3p, miR-210, miR-221, miR-222, miR-222-3p, miR-223, miR-320, miR-320e, miR-328-3p, miR-339-5p, miR-340-5p, miR-374-3p, miR-376a, miR-376b, miR-376c, miR-454, miR-485-3p, miR-486, miR-486-5p, miR-497, miR-543, miR-548b-5b, and RNU6-1 [19,62,63,64,65,66,67,68,69,70,71,72,73,74,75,76,77,78,79,80]. Upregulation of miR-21 may serve as an early diagnostic but also as prognostic [63] and monitoring marker [81], whereas panels of different miRNAs were found to be potential markers for diagnostics and tumor grade, as well as prognostics [82]. In an elegant new model, urine samples from mainly glioma patients and noncancer individuals were used to develop with artificial intelligence (AI) a diagnostic model for the detection of such tumors in urine samples [83]. A panel of 23 miRNAs was found to separate noncancerous from glioma patients. However, a common standard needs to be established and validated to diagnose tumor patients not only from noncancer individuals, but also from patients with other diseases, such as inflammations or degenerating diseases. Future studies may also include circular RNAs (circRNA) as possible markers. They are more stable than single-stranded RNA, and some of them can serve as a functionally antagonistic sponge for specific miRs and, therefore, are significantly involved in gene regulation [57].

**Table 3:** Examples of circulating miRNA markers in brain tumors.

Year	miR	Variation	Source	Method	Tumor
2016 [81]	miR-10-b	Up/progression	Serum	qPCR	HGG
	miR-21				
2016 [70]	miR-205	Down/diagnostics	Serum	qPCR	Glioma
2018 [67]	Panel of 7 miRNAs	Diagnostic signature	Serum EV	NGS	GBM
2020 [65]	miR-21	Up/progression	Serum	ddPCR	LGG, GBM
	miR-20e				
	miR-223				
2020 [66]	miR-17-5p	Up/progression	Serum	qPCR	GBM
	miR-125b				
	miR-221				
2020 [84]	miR-486	Up/diagnostic	EV from tumor microenvironment/neurosurgical aspirate fluid	NGS	GBM
2021 [85]	miR-21	Up/progression	Serum EV	qPCR	HGG
	miR124-3p				
	miR-222				
2021 [83]	Panel of 23 miRNAs	Screening signature	Urine	nanowire	GBM, glioma

### 2.4. Extracellular Vesicles—EVs

Tumor and normal cells can release small, extracellular vesicles into body fluids, such as blood and CSF. In addition to proteins, these vesicles contain DNA and RNA, including miRNA, which are protected by the cellular membrane. EVs can be analyzed to reliably detect tumor-specific mutations, including amplification of wild-type EGFR [86,87,88]. CSF appears to have an advantage over serum, perhaps due to the reduced number of EVs from leukocytes compared to blood. For example, IDH1 mutation G395A was detected in CSF-derived EVs of glioma grades II, III, and IV with a sensitivity of 63% and a specificity of 100%, but not in frozen serum [58]. Using quantitative PCR changes in wildtype IDH1 levels can also be used to monitor tumor burden and treatment response when the tumor does not have an IDH-1 mutation [58]. Most glioblastomas have an amplification of the wild-type epidermal growth factor receptor gene (EGFR), which results in an increased RNA expression; this amplification can be detected (indirectly) by quantitative reverse transcriptase-PCR (qRT) of CSF-derived EV RNA [86]. Using the same method, another typical mutation in glioblastomas can be detected, EGFRvIII, which lacks several exons. This deletion mutant was also detected in EV from blood in high-grade gliomas (III and IV) [87,88] and may serve as a good biomarker. EVs from serum or from neurosurgical fluid were also used to detect miRNAs from glioblastomas [67,84].

## 3. Discussion

**Figure 2:**
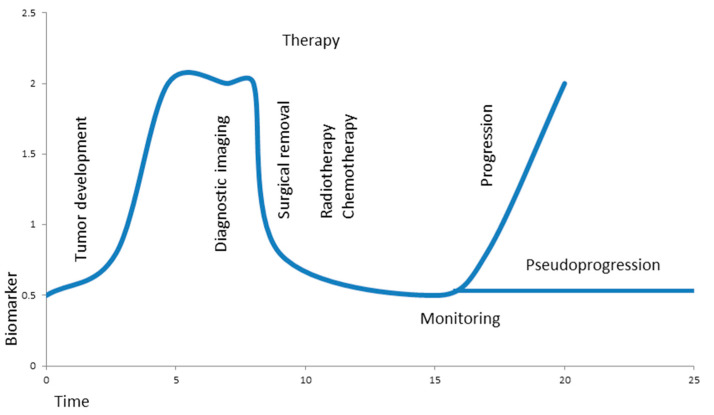
Hypothetical biomarker during brain tumor development, therapy, and monitoring. A growing tumor causes elevated levels of a biomarker at time of diagnostic imaging; the levels drop significantly after surgical removal of the tumor and stay low during additional radio- and chemotherapy. Liquid biopsy allows minimally invasive, real-time monitoring in order to differentiate progression from pseudoprogression of the tumor.

Over the past two decades, liquid biopsy has been developed from initial research studies up to reimbursed clinical applications in at least a few cancers, such as genetic monitoring of lung, breast, and prostate cancer and melanoma after therapies, but this is still quite far away from a real routine setting outside academic hospitals and most medical practices. During the last decade, almost all hopes for a fast improvement of liquid biopsy were disappointed. Although an emerging number of studies showed rather encouraging results for a whole range of tumor entities and for many clinical applications, e.g., diagnosis, prognosis, monitoring, and therapy decisions, there is still a major lack of standardization of methods and useful genetic markers; this appears to be true especially for brain tumors. It was quite surprising to find circulating brain tumor cells within the bloodstream only recently [27], suggesting their clinical utility for brain tumor patients. Therefore, established detection methods from molecular genetics and their use as diagnostic tools for the analysis of tissue samples were applicable to liquid biopsy in brain tumors. Encouraging results showed that liquid biopsy can be used also for brain tumors and should be further developed; currently, CSF as a source for liquid biopsy appears to be superior to blood-derived samples, serum, or plasma. Although both CSF and blood can be used to find CTCs, cell-free nucleic acids, EVs, and miRNAs, the use of CSF results in a much higher sensitivity. When collecting CSF, there seems to be a slight advantage of cisternal over lumbar puncture in detection sensitivity [89]; nevertheless, the individual risk of obtaining CSF has to be considered and damage to the brain must be avoided. Despite the typical mutations known from tissue biopsies, partly being relevant for therapeutic decisions, there is a chance for additional markers as candidates for liquid biopsy, including some splice variants of CD44, which can be found in glioblastomas, but often restricted to clusters of tumor cells in the tumor tissue [90]. Tumor-specific splice variants of CD44 may be worth testing for their potential ease of use in tissue and liquid biopsies (Eibl, unpublished). Detection limits vary and sensitivity appears to be better from CSF, but the risk for lumbar puncture, especially with a large tumor mass in the brain, is higher than for drawing blood. Data from very different clinical studies and approaches using liquid biopsy may be shared in fair conditions for reanalysis by others, similar to cell migration studies in publicly available data repositories [91]. It is likely that direct tumor sampling by removal of a brain tumor or stereotactic biopsy of tumor tissue will remain the method of choice to allow for reliable diagnosis by both histology and genetic profiling. However, in the near future, liquid biopsy as a potential alternative may be used in cases, where it is risky to get direct access to tumor tissue due to the location or due to comorbidities. Currently, the low incidence of brain tumors and the lack of evidence for early treatment options prevent the application of liquid biopsy in early tumor screening. There is more potential to use liquid biopsy in follow-ups of the patient; changing levels of biomarkers before and after treatments can be used for prognosis, monitoring of treatment outcome, and therapy decisions, while having a significantly lower risk than repeated stereotactic, surgical biopsies (Figure 2). With the new era of immune therapies also evolving for brain tumors, there is a need to have useful tools for monitoring tumor growth and the response to therapeutic treatments, as well as for prognostic evaluations [92]. This will allow clinicians to better support patients in their decisions at different stages of the disease. With now about two decades of increasing hope, but also many disappointments, liquid biopsy appears to be able to reach a new level of usefulness outside of just academic settings. Currently, however, there are limited applications of liquid biopsy which can be reimbursed for only a few non-brain tumor entities, such as lung, breast, and prostate cancer and melanoma. In the future, it is reasonable to expect a wider range of tumor entities to use liquid biopsy in one way or another. The isolation of CTCs appears to be a very promising approach, which may allow a better evaluation of the tumor stage than classical biopsy, although CTCs surely also do not represent the whole tumor. Unfortunately, brain tumors lack the epithelial markers used to isolate at higher efficiency CTCs from carcinomas. Rather, new methods may be worth investigating in living cells, including CTCs; for example, a pioneering approach with atomic force microscopy (AFM) on living tumor cells includes the functional, biophysical, and pharmacological characterization of individual tumor cells down to a so-called single-molecule level, as well as their pharmacology of chemokine and cell adhesion receptors and their regulation in real-time [93,94,95,96] (Eibl, unpublished). Cell-free nucleic acids, as well as extracellular vesicles and miRNAs, are, in principle, also really promising targets; however, again, there is a lack of established biomarkers, and a standard of methods first has to be developed with reliable, larger, clinical studies, especially for brain tumors. Future searches for feasible genetic markers may include brain tumor-specific splice variants of CD44, since they appear to be expressed on astrocytomas and glioblastomas and differ from known variants of leukocytes [90] (Eibl et al., unpublished). It may take time; however, for a restricted number of patients and brain tumor entities, for example, medulloblastomas in children, one may find a way to bring liquid biopsy to the level of routine standard in clinical settings.

Major data from clinical studies, even with negative results, are commonly available as publications and their supplements; sharing more data for reuse and meta-analysis may allow faster progress. For example, cell migration as a hallmark of glioblastoma cells invading other tissues may become a target for therapy by unlocking the complex biological processes of CTCs entering the blood or CSF; therefore, sharing big data in shared repositories and making them Findable, Accessible, Interoperable, and Reusable (FAIR) may allow computer scientists to find faster suitable markers or treatments [91].

Over a century after Harvey Cushing significantly contributed to modern neurosurgery, the clinical outcome of, e.g., glioblastoma, has not changed very much. With Gazi Yasargil, half a century ago, neurosurgery improved again, but there now seems to be only limited room left for improvement of highly skilled neurosurgeons, perhaps continuing to develop or modify their own instruments for sophisticated neuroanatomic findings [97] or for automated support from special microscopes. In addition, improving diagnostic imaging, e.g., MRI with increasingly stronger magnets, usually ranging from 1.5 T to 3 T, but rarely to 7 T and more, may allow much higher resolution and more applications. However, a leap forward appears to be a possibility with the liquid biopsy of brain tumors in combination with developing immune therapies. It hopefully may not take another century to clearly improve the clinical outcome of the most severe cases of brain tumors.

## 4. Conclusions

Over the following years, we expect clinical studies to present data on the different approaches in liquid biopsy that will provide the best value for patients with primary brain tumors. Most likely, personalized genetic profiling of a tumor from an initial tissue biopsy will indicate several possible markers to choose from for later use in liquid biopsy to monitor the treatment response. Such genetic markers show already close to 100% specificity [41,42,43,45]. The big challenge for integrating liquid biopsy into clinical routine will be to evaluate reliable standards and to increase the rather low and variable sensitivity from often only around 10–60% [40,44,45]. Sample volumes, technology improvement, and application of artificial intelligence will have to be optimized to increase the lower sensitivity. CSF samples appear to be often better than blood samples; however, with an improvement of technology, both approaches can be valuable. Urine samples may offer a potential for diagnostic screening of brain tumors with detecting panels of miRNA; however, since primary brain tumors are not common and treatment options are limited, diagnostic screenings may not come within the next decade. The authors are optimistic that liquid biopsy will improve the monitoring of disease and response to treatment in patients with brain tumors.

## Figures and Tables

**Table 1:** Pros and cons of liquid biopsy in brain tumors.

Factor	Source	Pro	Con
CTC	CSF, blood	Specificity +++ Molecular diagnosis, fast and easy monitoring of tumor growth and therapy response, may represent relevant part of tumor and be superior to local tissue biopsy	Sensitivity −−− Very rare, difficult to isolate, no standards established, may not represent whole tumor, more experimental studies needed
Cell-free nucleic acid (DNA, RNA)	CSF, Blood	Specificity +++ Molecular diagnosis, established methods, easier to collect than CTC, fast and easy monitoring of tumor growth and therapy response, may represent relevant part of tumor and be superior to local tissue biopsy	Sensitivity − May not represent whole tumor, no final standard established, depends on location of tumor near CSF
EV	CSF, blood, neurosurgical fluid (including DNA, RNA, microRNA)	Specificity +++ CSF better than blood, due to less background from leukocytes	Sensitivity − Background from normal cells (leukocytes), e.g., in blood
miRNA	CSF, blood, urine	Specificity +/− Potential for easy and specific therapy monitoring, perhaps diagnostic tumor screening, depends on selection of markers/panels	Sensitivity −−− No tumor specific sequences, needs normal reference, not standardized

Notes: +++—very high; +/−—moderate; −—low; −−−—very low.

**Table 2:** ctDNA markers tested in liquid biopsy of brain tumors.

Year	Gene	Variation	Source	Method	Tumor
2003 [39]	MGMT (promoter)	Methylation	Serum	MS-PCR	GBM
2006 [45]		Methylation	Plasma	MS-PCR	GBM, AA
2010 [41]		Methylation	Serum	MS-PCR	Astrocytic tumors (WHO III, IV), oligodendroglial tumors (WHO II, III)
2013 [42]		Methylation	Serum	MS-PCR	Glial tumors (II, III, IV), meningioma
2003 [39]	p16	Methylation	Serum	MS-PCR	GBM
2006 [45]		Methylation	Plasma	MS-PCR	GBM, AA, AOA
2003 [39]	DAPK	Methylation	Serum	MS-PCR	GBM
2003 [39]	RASSF1A	Methylation	Serum	MS-PCR	GBM
2013 [42]		Methylation	Serum	MS-PCR	Glial tumors (II, III, IV), meningioma
2006 [45]	p73	Methylation	Plasma	MS-PCR	GBM
2010 [41]	PTEN	Methylation	MS-PCR	MS-PCR	Astrocytic tumors (WHO III, IV)
2014 [40]		Mutation	Plasma, serum	Digital PCR, sequencing	Glioma II, AA, GBM
2010 [41]	10q	LOH	Serum	LOH	Astrocytic (WHO III, IV), Oligodendroglial (WHO II, III)
2010 [41]	1p	LOH	Serum	LOH	Oligodendroglial (WHO II, III)
2010 [41]	19q	LOH	Serum	LOH	Oligodendroglial (WHO II, III)
2012 [43]	IDH1	Mutation (R132H)	Plasma	digital PCR	Glioma (WHO grade II, III, IV)
2014 [40]		Mutation	Plasma, serum	Digital PCR, sequencing	Glioma II, AA, GBM
2013 [42]	p15INK4B	Methylation	Serum	MS-PCR	Glial tumors (II, III, IV), meningioma
2013 [42]	p14ARF	Methylation	Serum	MS-PCR	Glial tumors (II, III, IV), meningioma
2014 [40]	TP53	Mutation	Plasma, serum	Digital PCR, sequencing	Glioma II, AA, GBM
2014 [40]	EGFR	Mutations	Plasma, serum	Digital PCR, sequencing	Glioma II, AA, GBM
2014 [40]	PIK3CA	Mutation	Plasma, serum	Digital PCR, sequencing	Glioma II, AA, GBM
2015 [54]	TP53 (R114C) EPHB1 TERT PIK3CG IDH1 (R132H) ANK (K2337X) EGFR (C620S) PTEN (D162) FTH1 (R108K) OR51D1 (R135C)	Mutations	CSF, (plasma)	ddPCR, MAF	GBM
2015 [53]	Genome	Mutations	CSF	TAS/WES	AA III, PA I, ependymoma, medulloblastoma IV, GBM, LGG II, diffuse astrocytoma
2015 [52]	Gene panel (587 genes) including NF2, AKT1	Mutations	CSF, plasma, serum	ddPCR/TAS	Vestibular schwannoma, atypical meningioma
2017 [44]	Gene panels (54, 68, 70 genes) including p53, EGFR, Met	Mutations	Plasma	NGS	Brain tumors (not specified)
2018 [49]	IDH1, IDH2, TP53, TERT, ATRX, H3F3A, HIST1H3B	Mutations	CSF	sequencing	Diffuse gliomas
2018 [51]	Genome	SCNAs and fragmentation	CSF	WGS	Glioma
2018 [59]	TERT	Mutation	CSF, (plasma)	PCR, sequencing	GBM
2019 [60]	BRAF	Mutation (V600E)	CSF, plasma, serum	dPCR	PXA, ganglioglioma, PA, pilomyxoid astrocytoma
2019 [47]	Genome including TP53, JAK2, NF1, EGFR, BRAF, IDH1, NRAS, GNAS, ATM	Mutations	Plasma	NGS	Astrocytic/oligodendral tumors grades I–IV, including GBM, medulloblastoma, meningioma, and ependymoma
2019 [50]	IDH1 1P19Q CIC ATRX TP53	Mutations	CSF	NGS	LGG, GBM

Note: MS-PCR = methylation-specific PCR; AA = anaplastic astrocytoma; AOA = anaplastic oligoastrocytoma; GBM = glioblastoma multiforme; LGG = low-grade glioma; LDA = low density array; MAF = mutant allelic frequency; PA = pilocytic astrocytoma; PXA = pleomorphic xanthoastrocytoma; qRT-PCR = quantitative reverse transcriptase polymerase chain reaction; RNA = ribonucleic acid; WHO = World Health Organization tumor grading; I, II, III, IV = tumor grade I, II, III, IV (not necessarily identical to WHO grading); NGS = next generation sequencing; TAS = targeted analysis sequencing; WES = whole exome sequencing; WGS = whole genome sequencing.
